# Establishment and preliminary evaluation of CT-based classification for distal radius fracture

**DOI:** 10.1038/s41598-024-60416-9

**Published:** 2024-04-27

**Authors:** Jun Zhang, Xiaoke Yao, Yanan Song, Peng Yin

**Affiliations:** 1 Department of Orthopaedics, The Affiliated Hospital of Innermongolia Medical University, Hohhot, 010010 China; 2 Department of Orthopaedics, Chinese People’s Liberation Army General Hospital (301 Hospital), Beijing, 100038 China; 3 Department of Orthopedics, Chengdu First People’s Hospital, Chengdu, 610041 Sichuan China; 4 National Engineering Laboratory for Medical Big Data Application Technology, Chinese People’s Liberation Army General Hospital, Beijing, 100853 China; 5 Department of Orthopaedics, Beijing Chao-Yang Hospital, China Capital Medical University, Beijing, 100020 China

**Keywords:** Wrist fractures, Bone fracture, Classification, Outcomes research, Trauma

## Abstract

Establish a new classification system of distal radius fracture based on computed tomographic (CT), and evaluate its reliability and reproducibility preliminarily, and provide a new theoretical reference for clinicians to use the clinical classification system. The imaging data and clinical data of 204 inpatients with distal radius fracture during 6 years from January 1, 2014 to January 1, 2019 in orthopaedic department were analyzed retrospectively and classified based on CT. Three observers evaluated the image data of 48 randomly selected cases based on CT at different time nodes of T1 and T2. Cohen's kappa was used to calculate the consistency. At the last follow-up, patients' Disabilities of the Arm, Shoulder and Hand (DASH), Patient Rated Wrist Evaluation (PRWE), and VAS scores were collected. Among 204 cases, there were 12 cases of type 1, including 6 cases of type 1-D, 4 cases of type 1-V and 2 cases of type 1-R. There were 6 cases of type 2, including 2 cases of type 2-DV, 2 cases of type 2-DR and 2 cases of type 2-VR. There were 186 cases of type 3, including 32 cases of type 3–0, 127 cases of type 3–1 and 27 cases of type 3–2. There was no significant difference in DASH, PRWE and VAS scores among all types (*P* > 0.05). The results of interobserver reproducibility were kappa = 0.985, ICC = 0.984 in the first evaluation, kappa = 0.986, ICC = 0.986 in the second evaluation. The results of intraobserver reproducibility were O1 = 0.991, O2 = 0.991, O3 = 0.989 respectively. The new classification system of distal radius fracture based on CT has theoretical and practical significance for incision selection, fracture reduction and internal fixation. 123 classification system is clear, comprehensive, easy to understand and remember. Moreover, it has higher interobserver reliability and intraobserver reproducibility than other systems reported at present.

## Introduction

Fractures occurring at the distal end of the radius are seen frequently in emergency departments, representing approximately one-sixth of all fractures^1^. Fracture classification is helpful to analyze the severity, guide the treatment and judge the prognosis. The four classifications of distal radius are widely recognized, including AO/OTA classification (proposed in 2007)^2^, Frykman classification (1967 Proposed)^3^, Fernández classification (proposed in 1991)^4^ , Universal classification (proposed in 1993)^5^. Although the classifications are defined and described based on the different characteristics of each fracture, however, the severity of the fracture, post feature stability assessment, the prognosis judgment, and the guide of treatment cannot be adequately judged by these classifications.

The ulnar styloid process was firstly included in the classification by Frykman without consideration of the fracture comminution, the fracture displacement, and the radial shortening. Therefore, this classification is not practical for the treatment guide and the clinical prognosis judgment. The advantage of the Fernández classification is high practicability, predicting the stability of the wrist joint and related soft tissue damage after injury. It provides a treatment basis and reasonable recommendations for adults and children with fractures. However, the classification is complicated and time-consuming due to determining the classification in conjunction with the injury mechanism. The Universal classification only distinguishes between extra-articular and intra-articular fracture, displaced and non-displaced fracture. Although the AO classification is relatively comprehensive and covers various distal radius fracture morphologies as the preferred classification system for the judgment of the severity of the post-injury condition, guiding the treatment and the retrospective evaluation of clinical treatment. The classification systems are detailed and complicated, which are limited in the clinical application.

Numerous studies assess the observer-consistent results of the radial fracture classification system based on plain radiographs. Minority evaluation that the observer-consistent results of the AO/OTA type are entirely credible, the majority assesses that the observer consensus results for AO/OTA group, “Fernández," “Universal," which are moderately credible or below. Therefore, there is no golden standard or recommendation for the classification of distal radius fracture.

The purpose of this study is to establish a new classification system for distal radius fracture based on CT and to evaluate its reliability and repeatability.

## Materials and methods

### Patient information

A total of 419 inpatients were selected from the orthopedics department of the General Hospital of the People's Liberation Army from January 1, 2014, to January 1, 2019. We collected a total of 419 inpatients information, and the study included 204 patients. There were 66 males and 138 females with an average age of 57.25 ± 14 years (19–89 years). There were 114 cases on the left fracture and 90 cases on the right fracture. Surgeons did their examination firstly and evaluated the X-ray previously requested by the emergency team (physicians) and then they attempted closed reduction and applied a splint or cast and finally obtained a post reduction X-ray and CT, with a CT scan thickness of 5 mm. At the last follow-up, patients' Disabilities of the Arm, Shoulder and Hand (DASH), Patient Rated Risk Evaluation (PRWE), and VAS scores were collected. The minimum follow-up time is at least 1 year after injury.

*Inclusion criteria*

The age of patients ≥ 18 years.

*Exclusion criteria:*

1. Fracture of bilateral distal radius;
2. Multiple injuries; multiple upper limb fractures on the same side;
3. Fractures were accompanied by related nerve and blood vessel damage;
4. The patient's ipsilateral limb muscle motor system disordered before the fracture occurred, which affected the shoulder, elbow, and wrist joint disorders;
5. Patients suffered from wrist rheumatism, rheumatoid arthritis or wrist osteoarthritis before the injury;
6. previous wrist surgery;
7. Alzheimer's disease or other cognitive or mental disorders;
8. Open fracture;
9. Incomplete imageology data;
10. Patients refused to follow up.

### Classification introduction

**Figure 1:**
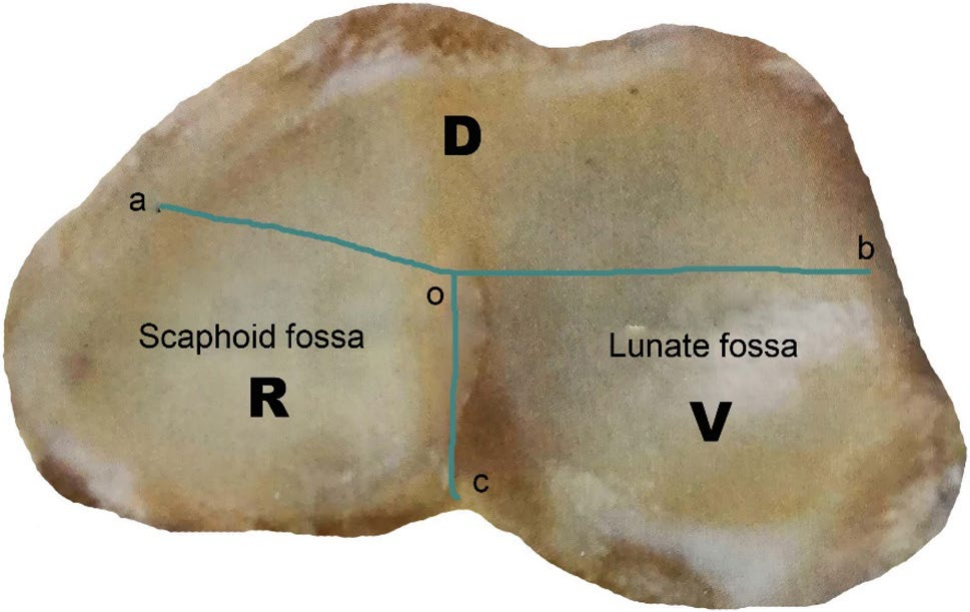
123 classification of distal radius fractures.

Point o was the midpoint of the longitudinal boundary between the scaphoid fossa and the lunar fossa; point a was the midpoint of the radial margin of the scaphoid fossa; With the o point as the starting point, the line along the junction of the scaphoid fossa and the lunar fossa, which was linked to the palmar margin as oc. The three lines oa, ob, oc divided the distal radial articular surface into three parts, namely the dorsal part -D, the palmar part -V, and the radial part -R. A part of the articular surface and cortex was separated from the radial shaft, which was defined as a fracture. Based on three parts, we divided the distal radius fracture into three types: single-part fracture (type 1), two-part fracture (type 2), and three-part fracture (type 3), namely type 1, 2, and 3. A single-part fracture can be D, V, or R. The single-part fracture represented the same meaning as the Arabic numerals “1”, which used the Arabic numerals "1"- type 1. The two-part fracture was D + V, D + R or V + R. The two-part fracture represented the same meaning as the Arabic numerals “2”, which used the Arabic numerals "2"- type 2. The three-part fracture involved D, V, and R at the same time. The three-part fracture represented the same meaning as the Arabic numerals “3”, which used the Arabic numerals "3"- type 3. In order to quickly understanding and memory, 123 classification were abbreviated. Type 1 included three subtypes of 1-D, 1-V, and 1-R; type 2 included three subtypes of 2-DV, 2-DR, and 2-VR, and type 3 included three subtypes of 3–0, 3–1, and 3–2. Three subtypes of type 1: 1-D: dorsal part fracture, 1-V: palmar part fracture, 1-R: radial part fracture. Three subtypes of type 2: 2-DV: dorsal part + palmar part fracture, 2-DR: dorsal part + radial part fracture, 2-VR: palmar part + radial part fracture. Type 3 was a three-part fracture; the three-part bone cortex was separated from the radial shaft cortex. According to the radial sigmoid fracture line, it was divided into three subtypes: 3–0: complete radial sigmoid notch, 3–1: 1 fracture line involved to the sigmoid notch, 3–2: ≥ 2 fracture lines involved to the sigmoid notch (Fig. 1).

### Observer consistency assessment

Three doctors were selected as observers, one orthopedic resident O1 with two years of work experience, one orthopedic physician O2 with 10 years of work experience, and one orthopedic deputy chief physician O3 with 25 years of work experience. The annual operation volume was more than 300 cases. Although the increase of the number of observers and included observation samples can make the evaluation results more accurate, the enlargement of included observation samples affected the accuracy of the evaluation results. Three doctors were included as observers as the previous study^6–8^. Three observers at the two different time points T1 and T2 used 123 typing in order to evaluate the randomly selected 48 cases imageology data according to the random number table method. The evaluation interval was at least three weeks. T1 node: 3 observers classified based on the DR and CT of the orthopedic wrist joint preoperatively according to the 123 classification system. T2 node: Three observers reclassified the anterior and lateral wrist joints DR and CT according to the 123 classification system before surgery. The image data of all patients were hidden, and the order of the image data of each case at each of the four nodes was randomized. Because it was a new type, the three observers studied and discussed together before the evaluation. Each time the observer types, another person was dedicated to recording.

### Statistical analyses

All data processing in this study was analyzed by R language software. Quantitative data were described using ${\overline{\text{X}}}$ ± S, and qualitative data were described using percentages. Multivariate analysis of variance was used to compare the DASH, PRWE, and VAS scores between different types. *P* < 0.05 was considered statistically significant. Cohen's kappa was used to calculate consistency. Cohen's kappa can be used to determine the consistency between two or more observers.

### Ethics approval and consent to participate

The study was approved by the Ethical Review Boards of the Chinese PLA General Hospital committee (Beijing, China), and was performed in accordance with the ethical standards of the Declaration of Helsinki of 1964. All participants provided informed consent before their participation in the study and written were obtained from all participants.

## Results

From January 1, 2014, to June 31, 2018, 419 inpatients information were collected according to inclusion and exclusion. 5 patients were treated with external fixation, 11 patients were treated with distal radius fractures, one patient was dementia, two patients were multiple injuries, and 1patient was open fracture. There were old fractures of the distal radius in 4 cases. Ten cases were bilateral distal radius fractures, 10 cases were with multiple fractures of the ipsilateral limb, 145 cases were with incomplete imageology data, and 19 cases lost to follow-up (3 of them died). Finally, 204 patients were included in the study. The general characteristics of the patients were shown in Table 1. Different classification results were shown in Table 2.

**Table 1:** Characteristics of patients (n = 204).

Extent of fracture involvement	Range		123 Type	Number (n)	Female (n, %)	Age (year)	Low energy damage (n, %)	Weight (kg)	Height (cm)	BMI (kg/m)	Smoke (n, %)
Type 1 (Single-part fracture)	**D**		1-D	6	4, 66.67	55.17 ± 19.91	6, 100	68.92 ± 16.33	167.67 ± 14.09	24.20 ± 2.60	1, 16.67
	**V**		1-V	4	2, 50	54.75 ± 19.72	2, 50	74.75 ± 9.54	170.25 ± 6.55	25.68 ± 1.68	0, 0
	**R**		1-R	2	0, 0	36.50 ± 3.54	2, 100	76.00 ± 8.49	176.50 ± 2.12	24.45 ± 3.32	0, 0
Type 2 (Two-part fracture)	**D + V**		2-DV	2	2, 100	66.5 ± 4.95	2, 100	53 ± 9.90	162.5 ± 7.78	20 ± 1.84	0, 0
	**D + R**		2-DR	2	1, 50	57.5 ± 2.12	1, 50	62 ± 18.38	162.5 ± 3.54	23.3 ± 5.94	1, 50
	**V + R**		2-VR	2	1, 50	44 ± 26.87	1, 50	65.5 ± 20.51	171 ± 8.49	22.1 ± 4.81	1, 50
Type 3 (Three-part fracture)	**D + V + R **	0	3–0	32	21, 65.63	59.94 ± 12.65	20, 62.50	67.52 ± 11.52	163.09 ± 9.66	25.27 ± 2.98	2, 6.25
		1	3–1	127	86, 67.72	56.99 ± 13.91	94, 74.02	66.75 ± 13.03	164.61 ± 8.99	24.49 ± 3.27	10, 7.87
		≥ 2	3–2	27	21, 77.78	57.89 ± 13.72	18, 66.67	64.22 ± 11.73	164.33 ± 5.87	23.69 ± 3.34	2, 7.41

**Table 2:** Comparison of 123 classification results.

Extent of fracture involvement	Range		123 Type	Number (n)	DASH (${\overline{\text{X}}}$ ± S)	PRWE(${\overline{\text{X}}}$ ± S)	VAS (${\overline{\text{X}}}$ ± S)
Type 1 (Single-part fracture)	**D**		1-D	6	4.58 ± 3.53	1.75 ± 1.54	0.33 ± 0.82
	**V**		1-V	4	2.9 ± 2.39	1 ± 0.82	0 ± 0
	**R**		1-R	2	0.4 ± 0.57	0.25 ± 0.35	0 ± 0
Type 2 (Two-part fracture)	**D + V**		2-DV	2	7.95 ± 1.77	2.5 ± 0	0 ± 0
	**D + R**		2-DR	2	5.4 ± 2.97	3.5 ± 4.24	1.5 ± 2.12
	**V + R**		2-VR	2	4.55 ± 5.30	3 ± 0	0 ± 0
Type 3 (Three-part fracture)	**D + V + R**	**0**	3–0	32	5.73 ± 3.69	3.40 ± 2.85	0.38 ± 0.66
		**1**	3–1	127	5.10 ± 3.78	2.40 ± 2.60	0.25 ± 0.72
		** ≥ 2**	3–2	27	5.80 ± 3.66	2.85 ± 2.38	0.22 ± 0.70
Not parting				0	–	–	–
F				0	0.9567	1.0491	1.0328
P				0	0.4712	0.4007	0.4127

The inter-observer reliability results were evaluated for the first time Kappa = 0.985, ICC = 0.984, and the second evaluation Kappa = 0.986, ICC = 0.986 (Table 3). Intra-observer reproducible results were Kappa for residents O1 of 0.91, Kappa of O2 attending physician 0.999, and Kappa of O3 associate chief physician 0.989 (Table 4).

**Table 3:** Inter-observer reliability results.

	Kappa value	ICC
The first assessment	0.985	0.984
The second assessment	0.986	0.986

**Table 4:** Intra-observer reliability results.

	Cohen’s Kappa
O1	0.991
O2	0.991
O3	0.989

### 123 classification system characteristics

The distal radius was divided into a dorsal part, a palmar part, and a radial part. The particular biomechanical function was in each portion corresponding to the injury mechanism (Table 5).

**Table 5.** 123 classification description of CT-based distal radius fractures.

Type	Range	123 Type	Characteristic	Injury mechanism	Surgical approach	Fixed form	
Single-part fracture 1	**D**	1-D	Fracture of the dorsal facet of the cranial fossa or combined with the dorsal facet of the navicular fossa. The articular surface and dorsal cortex cause syntripsis and subluxation or dislocation of the wrist	Dorsiflexion	Dorsal	Dorsal plate	
	**V**	1-V	Shear fractures of the volar articular surface of the cranial fossa, most of which are intact. subluxation or dislocation of the wrist is prone to cause	Palmar flexion	Palmar	Buttress plate	
	**R**	1-R	Radial styloid process shifts distally and ulnaris by avulsion violence; radial + stylus process shifts proximally and rotationally by bending + compression violence	Tear off or bend + compress	Radial	Radial styloid plate and screw	
Two-part fracture 2	**D + V**	2-DV	The lunar bone hits the lunar fossa, and the lunare articular surface is separated, and the fracture line passes through the sigmoid notch. Reconstruction of the sigmoid notch and check of TFCC stability are needed during the operation	Axial direction	Palmar	Palmar plate	
	**D + R**	2-DR	Radial styloid process + dorsal articular surface of scaphoid fossa or (and) dorsal articular surface of lunare fossa	Dorsiflexion + radial deviation	Palmar or dorsal part	Palmar plate or dorsal plate + radial plate	
	**V + R**	2-VR	Radial styloid process + fracture of the volar articular surface of the lunar fossa	Palmar + the axial violence	Palmar	Palmar plate	

Type	Range		123 Type	Characteristic	Injury mechanism	Surgical approach	Fixed form
Three-part fracture 3	**D + V + R**	**0**	3–0	Three-part division, complete sigmoid notch	The bending viokenve	Palmar	Palmar plate
		**1**	3–1	The three parts are split with the backbone cortex, which can be accompanied by a bone loss to varying degrees, and the radial sigmoid fracture line is 1	Axial violence predominates	Palmar, dorsal is united when it is necessary	Palmar plate + dorsal plate
		** ≥ 2**	3–2	The three parts are split with the backbone cortex, which may be accompanied by a bone loss to varying degrees, involving two or more sigmoid fracture lines of the radius	Axial violence predominates	Palmar, dorsal is united when it is necessary	Palmar plate + dorsal plate, bone graft when it is necessary

**Figure 2:**
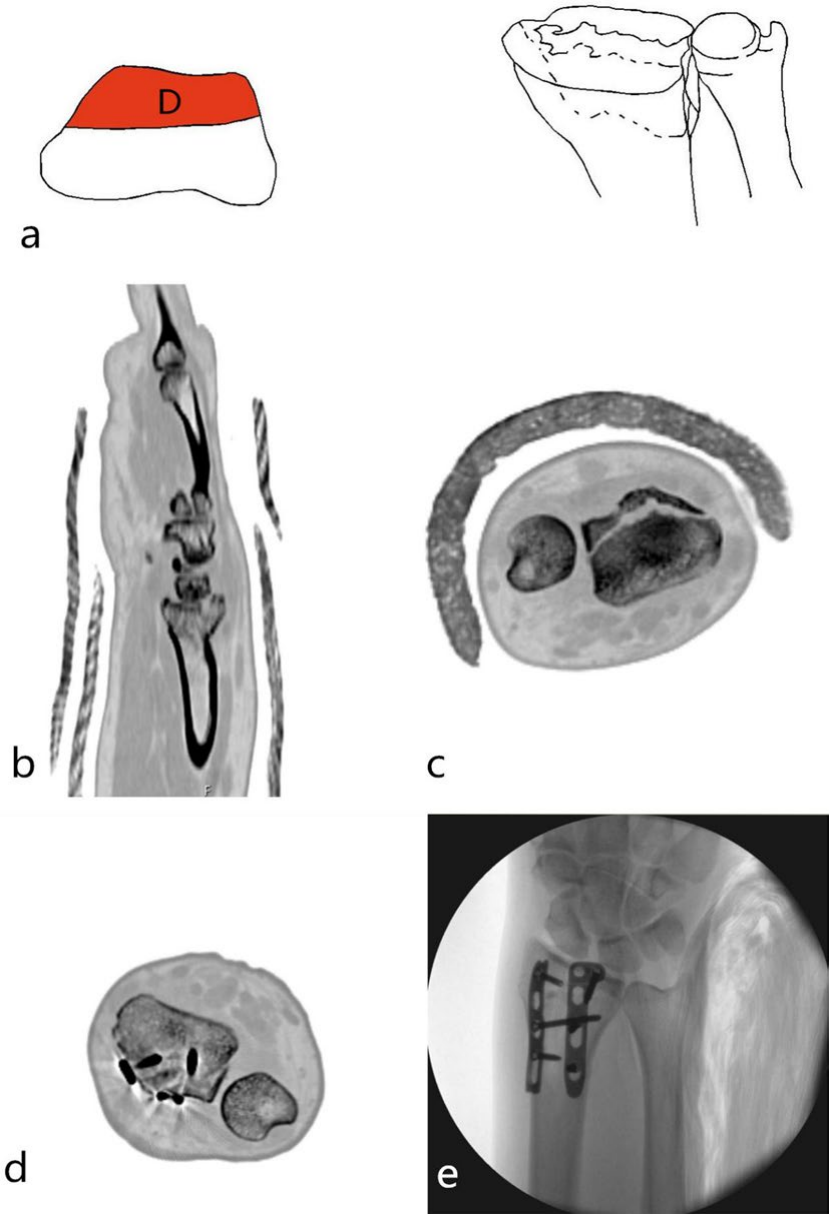
Distal radius fracture 123 classification A1.

Single dorsal part fracture (1-D): Separate dorsal part fracture was not common in type 1. It was 2-DV type fracture that the fracture involved both the dorsal and palmar parts. It was a 2-DR type fracture that the fracture involved both the dorsal and radial parts. Separate dorsal part fracture (type 1-D) was characterized by a dorsal fracture of the cranial fossa articular surface or combined with a fracture of the dorsal articular surface of the scaphoid fossa. The articular surface and dorsal cortex were comminuted fractures. It was prone to cause subluxation or dislocation of the wrist. The mechanism was wrist dorsiflexion injury, and the only surgical approach was the dorsal approach with the dorsal plate fixation. Poor restoration and fixation of the dorsal column fracture resulted in malunion, which eventually caused a decrease in the anatomical compatibility of the radial wrist joint and a malunion of the radial sigmoid notch (Fig. 2).

**Figure 3:**
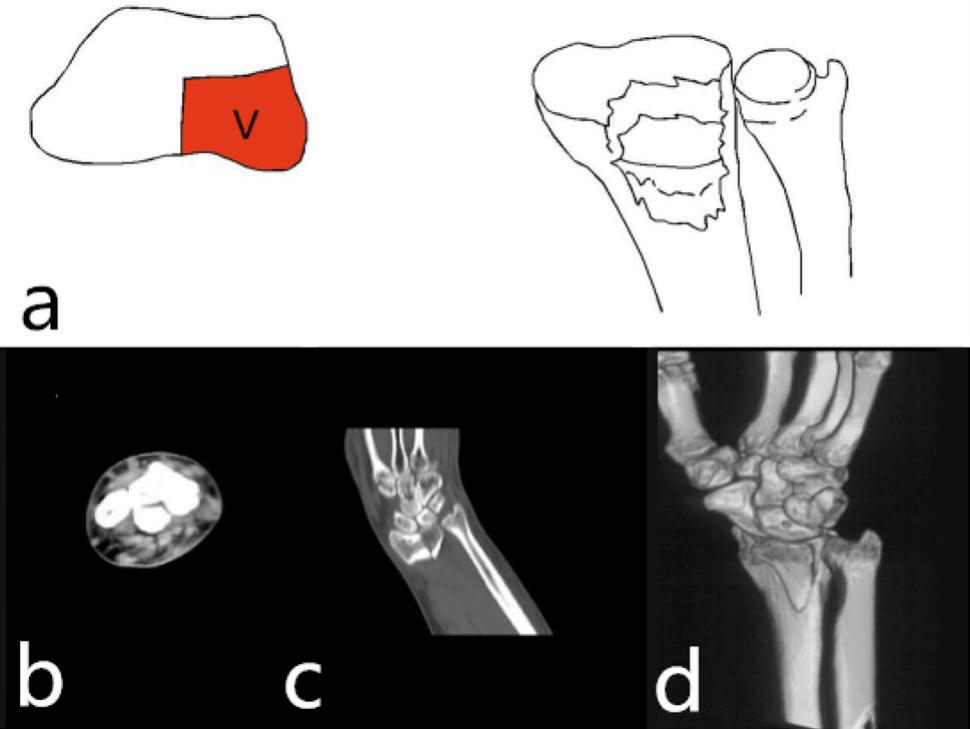
123 classification of distal radius fracture A2.

Single palmar part fracture (1-V): The fracture was characterized by a shear fracture of the palmar articular surface of the cranial fossa. Because the palmar cortex was strong and thick, the palmar cortex was mostly intact and rarely comminuted. Due to the tension of the stable radial wrist ligament, it can cause subluxation or dislocation of the wrist joint^9^. The mechanism was wrist palmar flexion injury. The only surgical approach for restoration and fixation was the palmar approach. The palmar support plate was selected for fixation. Especially when the palmar columnar bone was small, it was necessary to pay attention to the stable fixation of the fracture block to avoid secondary displacement, which may lead to subluxation of the proximal carpal bone (Fig. 3).

**Figure 4:**
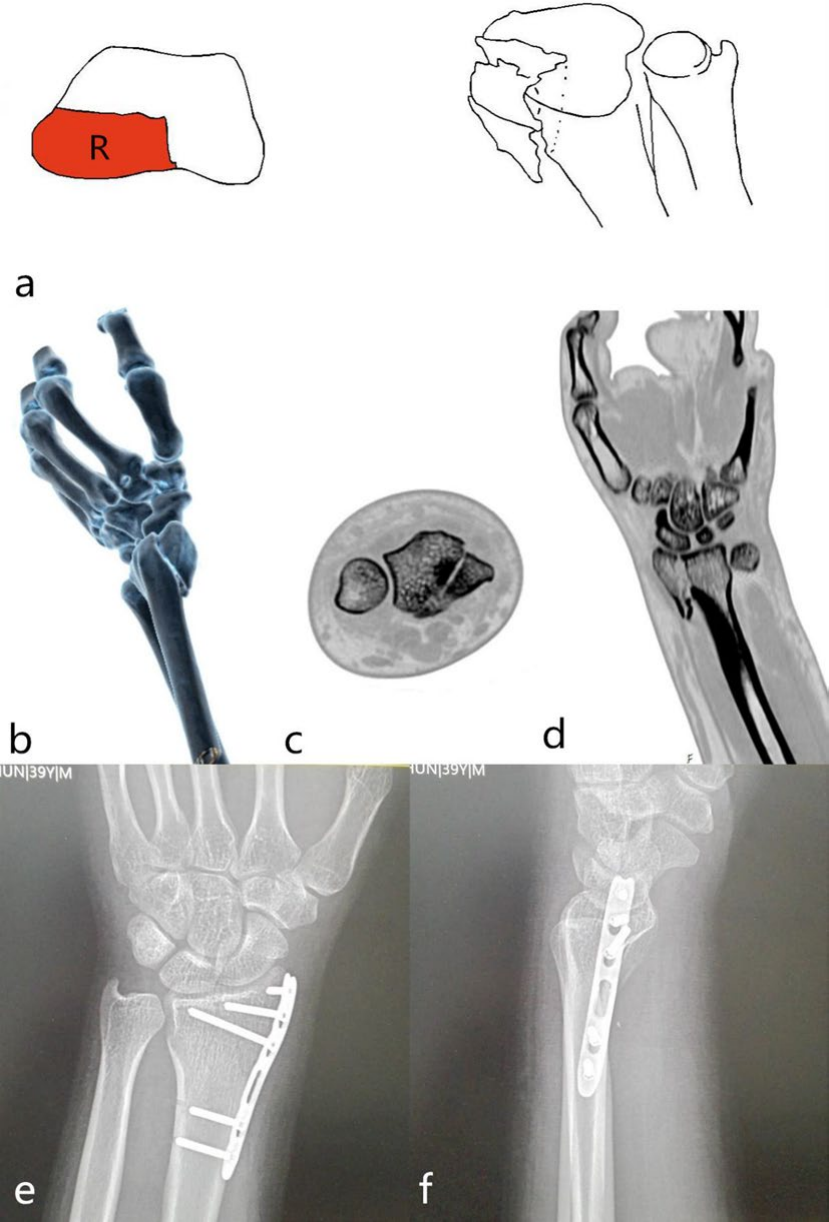
Distal radius fracture 123 classification A3.

Single fracture of the radial part (1-R): The radial part was composed of the radial styloid process and the palmar hem of the scaphoid fossa. A tough radial wrist ligament was attached around these structures. These structures ensured the flexible movement of the wrist with stability. The fracture was characterized by avulsion violence, causing the radial styloid process to shift to distal and ulnaris sides; While bending + compression violence was the leading cause, radial styloid process was shifted to intact side and rotation displacement due to the pull of the brachioradial muscle. Steps appear in radiocarpal joints. The injury mechanism is avulsion or bending + compression violence. The only surgical approach for reduction and fixation is the radial approach. The processus styloideus radii plate or screw was selected for fixation (Fig. 4).

**Figure 5:**
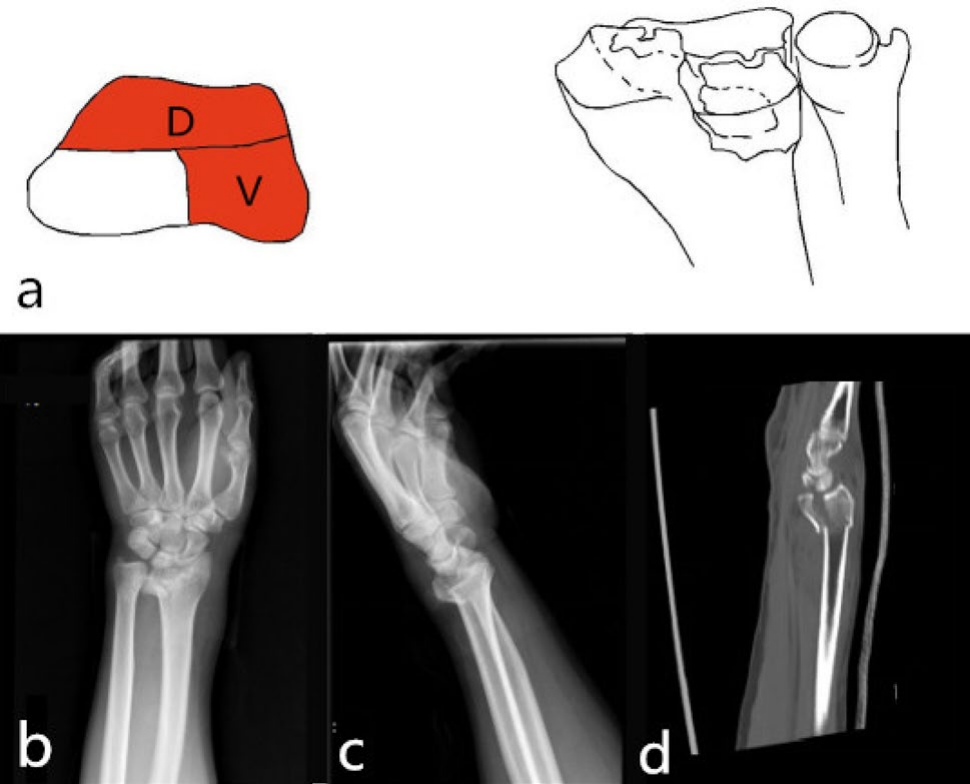
Distal radius fracture 123 classification B1.

Dorsal part + palmar part (2-DV): The fracture was characterized by the separation of the lunar bone articular surface caused by the collision of the lunar bone with the lunar bone fossa, and the fracture line involved the sigmoid notch. Reconstruction of the sigmoid notch and check of TFCC stability was necessary during the operation. The injury mechanism was the axial violence of the lunar bone striking the lunar fossa. The surgical approach for reduction and fixation was the palmar approach, and the palmar plate was selected for fixation. There was a rotation deformity of the palmar part and the dorsal part on the deformed surface; it was often the counterclockwise rotation of the palmar part and the clockwise rotation of the dorsal part, which led to the narrowing of the joint surface of the radial wrist joint. Part of the flexion function was lost; it was necessary to correct the rotational deformity during surgery^10,11^ (9, 10) (Fig. 5).

**Figure 6:**
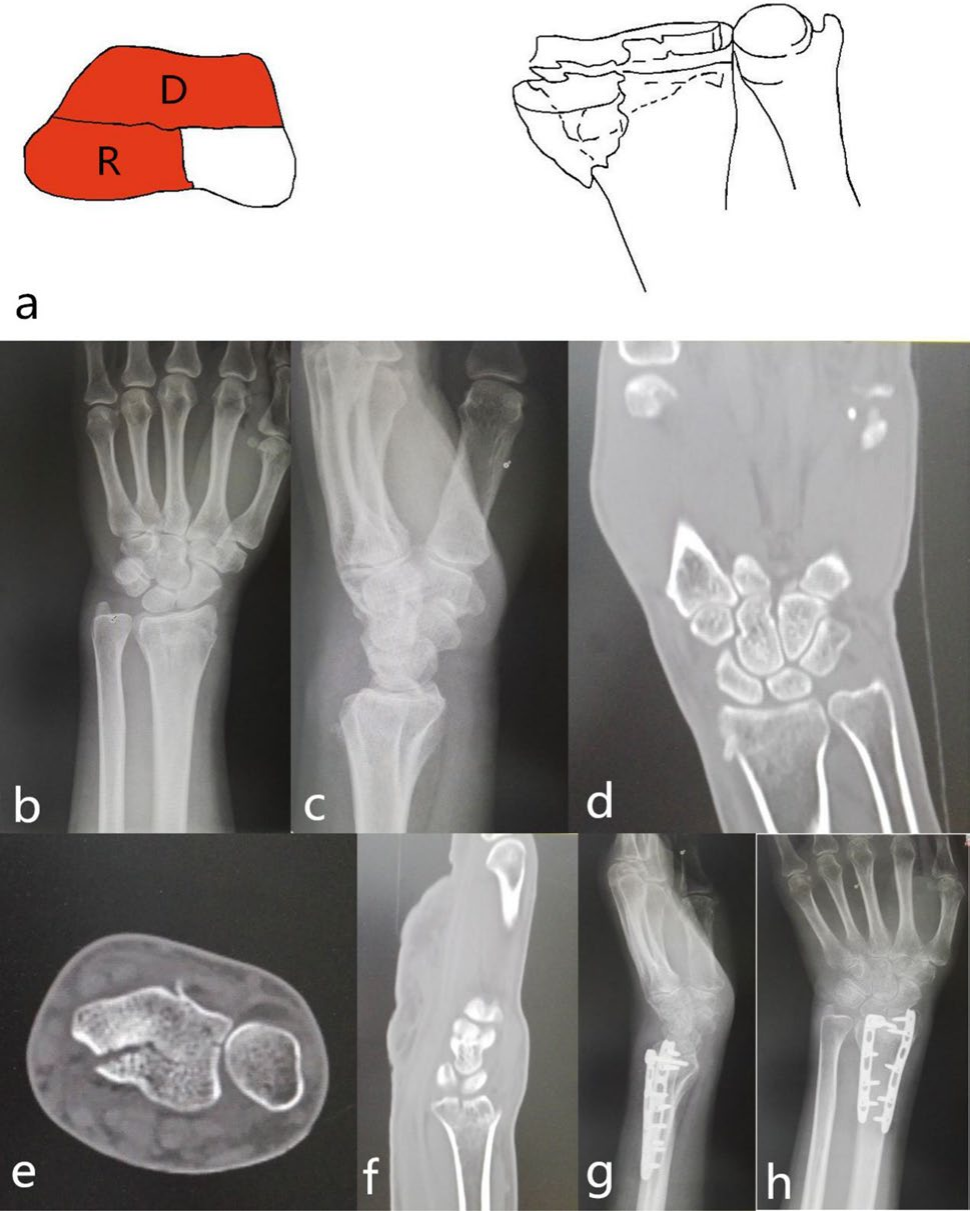
Distal radius fracture 123 classification B2.

Dorsal part + radial part (2-DR): The fracture was characterized by radial styloid process + the dorsal part of the scaphoid fossa or (and) the dorsal part of the lunar fossa. The injury mechanism was wrist palmar flexion injury; the incision was the palmar or dorsal approach, palmar support steel plate or dorsal + radial steel plate was selected for fixation. While the dorsal approach was used for fixation, the ulnar side of the Lister tubercle was separated, the third fascial sheath was exposed, and the fractured end was exposed. A steel plate was placed on the ulnar side of the Lister tubercle and screwed. Another plate was placed on the radial side of the Lister tubercle (Fig. 6).

**Figure 7:**
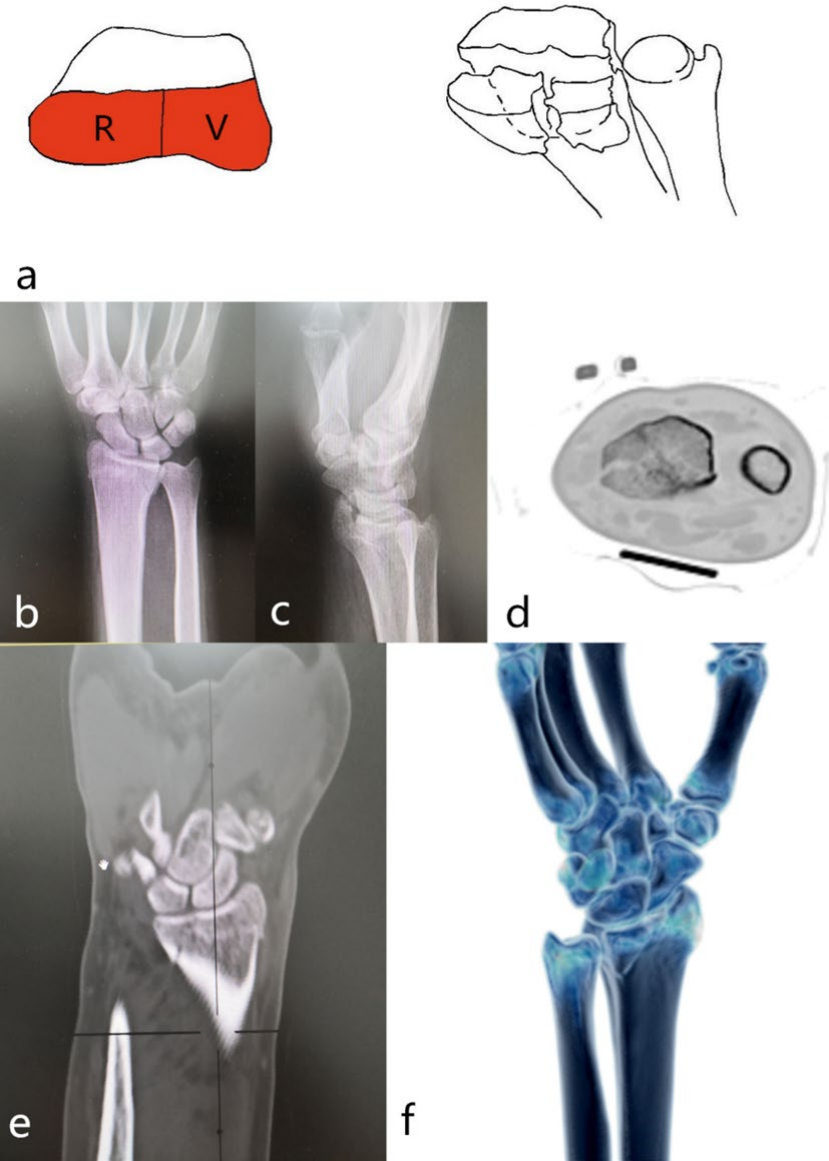
Distal radius fracture 123 classification B3.

Palmar part + radial part (2-VR): The fracture was characterized by radial styloid process + volar part fracture. The injury mechanism was wrist flexion injury. The surgical approach for reduction and fixation was the palmar approach. The palmar support plate was selected for fixation. (Fig. 7).

**Figure 8:**
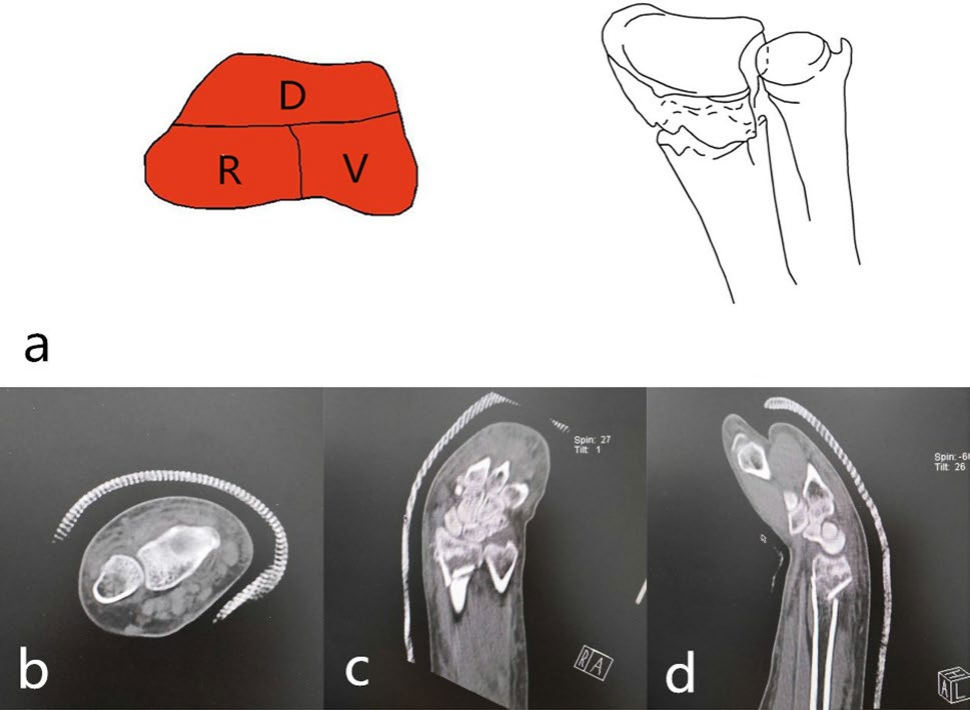
123 classification of distal radius fracture C1.

**Figure 9:**
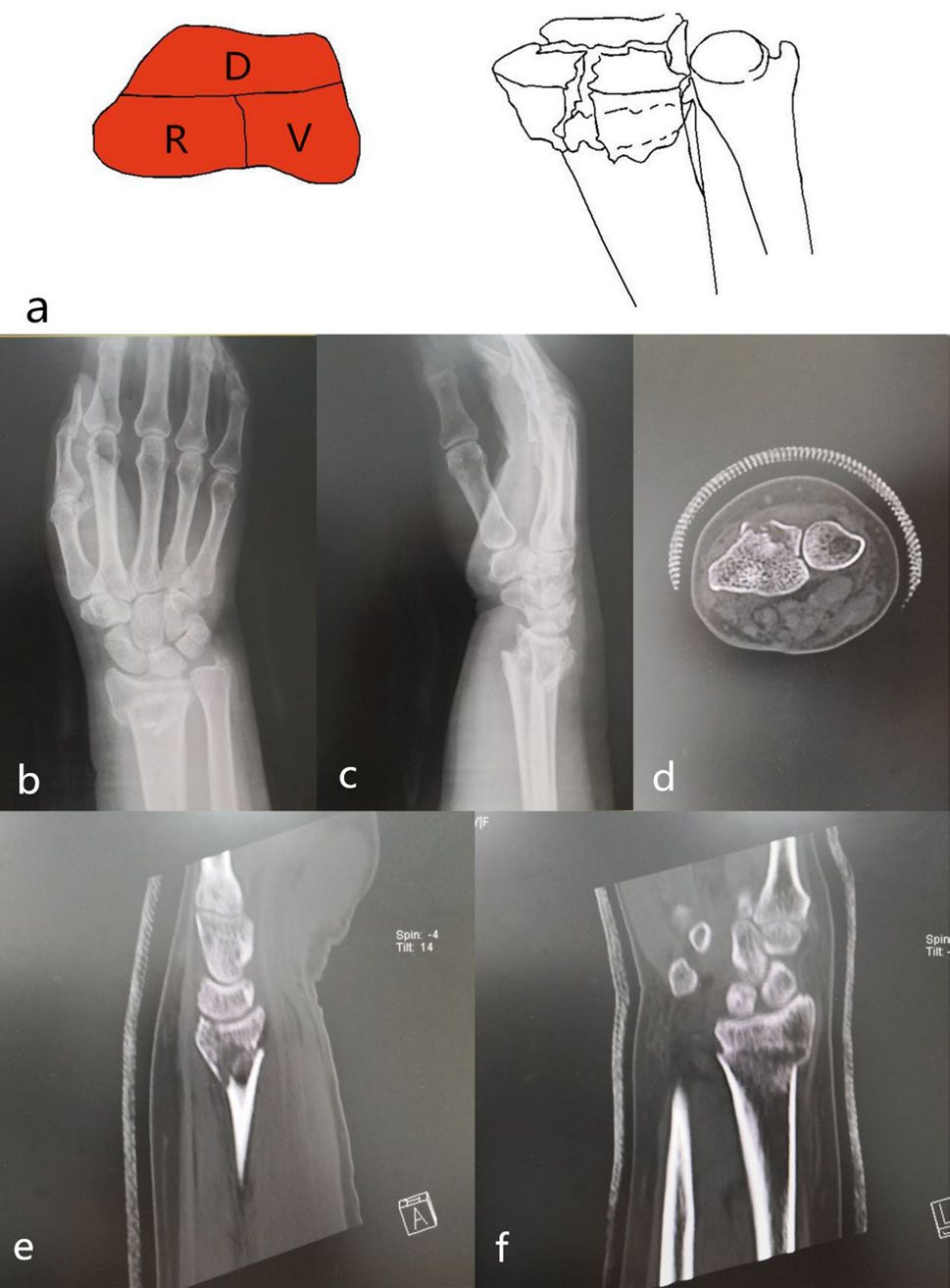
123 classification of distal radius fracture C2.

**Figure 10:**
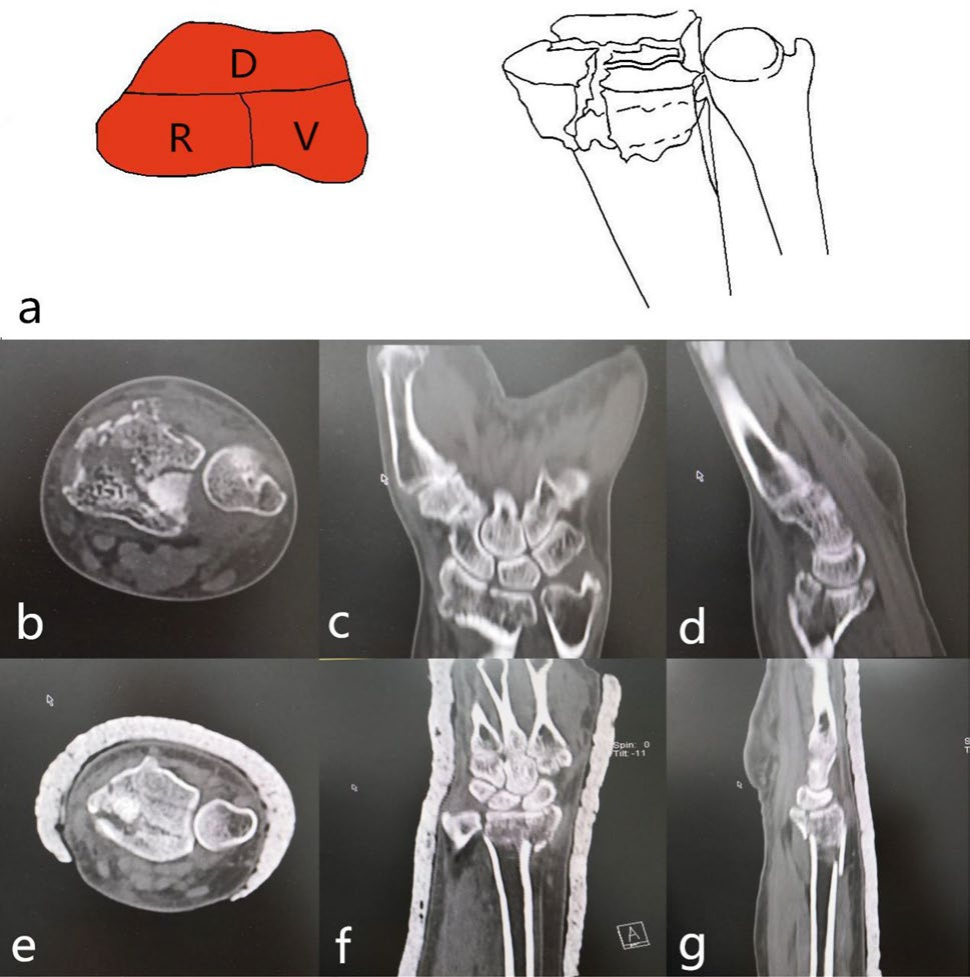
Distal radius fracture 123 classification C3 (b-d is case 1, e–g is case 2).

Dorsal part + palmar part + radial part (type 3): The fracture was characterized by three parts of the cortex separated from the radial shaft cortex, with varying degrees of bone loss. It was divided into three subtypes according to the radial sigmoid fracture line. Subtypes: 3–0: complete radial sigmoid notch, 3–1: 1 fracture line involving sigmoid notch, 3–2: ≥ 2 fracture lines involving sigmoid notch. The injury mechanism was bending or axial violence. 3–0 was an extra-articular fracture, the reduction and fixed incision of 3–1, 3–2 type was palmar approach, supplemented by a dorsal approach when it was necessary. It was first considered about placing the steel plate on the palmar about fixation. It was combined with the dorsal steel plate to assist in fixing as occasion required (Figs. 8, 9, 10).

## Discussion

In this study, the distal radius fracture was divided into three types: 1, 2, and 3 based on CT. Each type was divided into three subtypes. The patients included in our hospital were followed up. There was no significant statistical difference in each type of DASH, PRWE, and VAS scores. The reasons may include the following aspects. First, it was a selection bias of the included patients. This study included inpatients for inpatient surgery without including outpatient conservative treatment. Some inpatient datas were inadequate. Second, the wrist joint was a functional joint rather than a weight-bearing joint. There was little impact on the patient's daily functional requirements about the loss of the smaller range of the wrist joint. Therefore, the DASH, PRWE scores were low, which reflected the subjective functional outcome of a patient. However, the observer consistency evaluation results of the new typing system were more significant than 0.9, and according to the Landis and Koch standards, they were utterly credible. Moreover, the intra-observer repeatability results of the 123 classification were unrelated to the observer's experience.

### Requirements for an ideal classification system

A practical and widely accepted classification was characterized by the following characteristics: 1. Better observer-consistent results. 2. Characteristics of fractures can be accurately understood and described, the fracture injury mechanism can be reproduced, preoperative plans were formulated, treatment guidance was provided, and possible complications were accurately judged for clinicians. Moreover, it can be used as a credible “common language” for clinicians in different countries and regions. 3. It was comprehensive and easy to remember. Based on the above characteristics and requirements, we established an ideal 123 classification of CT-based distal radius fractures.

### Insufficient research on current conventional classification systems

At present, the observer-consistent results of the AO/OTA classification were entirely credible or basically credible in individual studies^12^. The majority of studies reported observations of AO/OTA group, Frykman type, Fernández type, and Universal type were moderately reliable or below^7,8,13–22^. All the above classifications were based on ordinary understandable film classification. The number and shape of fracture lines in the joint and the direction of fracture block displacement were diagnosed by CT. It was helpful to the classification understanding and a surgical plan. However, was it possible to improve observer-consistent results? At present, there were only two studies on whether the increase of CT examination can improve the observer consensus results of AO/OTA typing, Frykman typing, Fernández typing, and Universal typing^23,24^. The results of both studies were different. In 2014, research by Arealis and others indicated that adding CT examinations can help senior doctors to formulate treatment plans, but it cannot improve the observer-consistent results of AO/OTA, Fernández, and Universal classification^23^. In 2017, Kleinlugtenbelt et al. reported that except Frykman typing, the addition of CT + 2D examination could improve the repeatability within the observer of AO/OTA classification, Fernández typing, and Universal classification than using plain X-ray films alone, and it cannot improve all Inter-observer reliability of classification^24^. Our study found that adding CT + 2D examination did not improve observer-consistent results for Frykman classification, Fernández classification, and Universal classification. However, a noticeable improvement was in the observer consensus results of AO/OTA classification and AO/OTA grouping.

### Recent research on new typing system for distal radius

In view of the low reproducibility of the classification of distal radius fractures, Bellott et al. proposed an IDEAL classification method based on epidemiological and imaging factors^25^. Kapppa between observers of IDEAL classification = 0.595, and Kapppa within observers = 0.771. Yi Lu et al. described a morphological typing and grading system specifically for Barton fractures^26^. It was divided into four types by the research team: classic Bartong fracture, ulnar Bartong fracture, radial Bartong fracture, comminuted Bartong fracture, and the inter-observer reliability and intra-observer repeatability scores were 0.71–0.80 and 0.68- 0.88^26^.

A majority of literature pays attention to the radial wrist joint, while the distal radioulnar joint was ignored. Frykman's study indicated that the fracture line involved the inferior ulnar radial joint in 19% of cases^3^. Malunion of the distal radioulnar and weak line of force can lead to chronic pain and weaken of the wrist. Jupiter et al. found that distal radioulnar joint mismatch gradually developed arthritis, with varying degrees of wrist pain in 33% of patients^27^. Later, some scholars were classified according to the situation of sigmoid notch. Rozental et al. included imaging data of 20 intra-articular fractures. 13 (65%) intra-articular fracture lines extended to the sigmoid notch in CT data^28^. By the sigmoid notch image data analyzation, all cases were divided into three types, type 1 was the complete sigmoid notch, type 2 was fracture line involving sigmoid notch but no displacement, and type 3 was fracture line involving B The notch is shifted. Nakanishi et al. classified intra-articular fractures of the distal radius into type 3 and 5 subtypes based on the number of fracture lines involving the sigmoid notch and the degree of displacement of the fractured mass^29^. Their study found that sigmoid fractures were presented in 83% of cases of intra-articular fractures and that sigmoid fractures were comminuted in 34%^29^. The application of 3D-CT provided accurate judgment of the sigmoid notch damage and a basis for surgical strategy. Hintringer et al. combined the biomechanical foundation of the distal radius, a new classification based on CT has been proposed^30^.

### Theoretical basis and a basis for the 123 classification system

The theoretical basis for the establishment of the 123 classification system is based on the characteristics of the fracture line of the articular surface of the distal radius fracture, the bone ligament unit theory, the three-column classification of Rikli and Regazzoni^31^ and the four-part classification of Melone and many other theoretical studies^32,33^.

**Figure 11:**
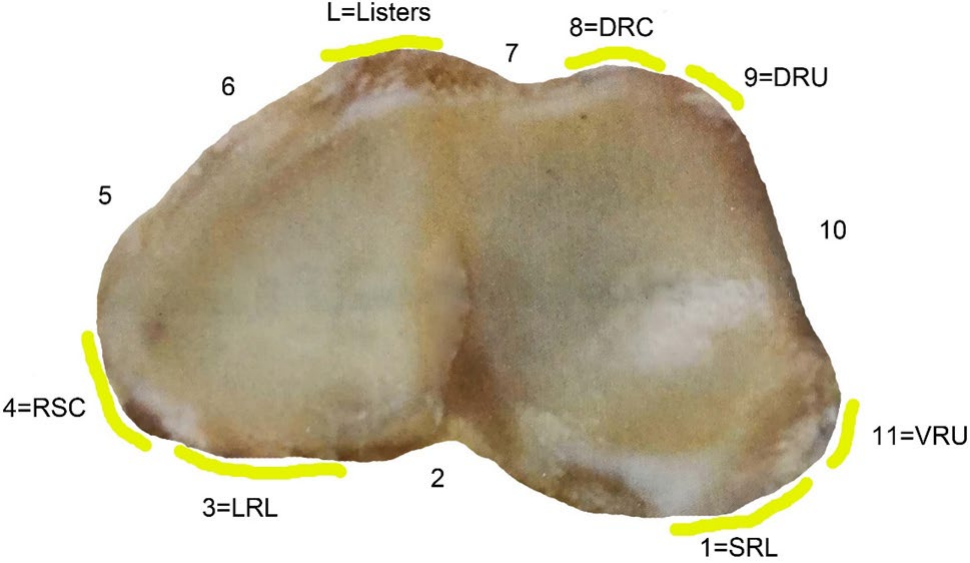
1. Short radiolunate ligament (SRL) 2. Radioscapholunate ligament (RSL) 3. Long radiolunate ligament (LRL) 4. Radioscaphocapitate ligament (RSC) 5.6.7. The area between the scaphoid ligament and the dorsal radiocarpal ligaments (DRC) is divided into three regions of 5.6.7 8. The dorsal radiocarpal ligaments (DRC) 9. Dorsal radioulnar ligament (DRU) 10. Radial sigmoid notch 11. Palmar radioulnar ligament (volar radioulnar ligament (VRU).

There are many ligament attachment points around the articular surface of the distal radius, and the fracture line often occurs between the ligament attachment areas with a certain regularity. Increasing evidence suggests that even if the distal radial articular surface was a comminuted fracture, the ligament attachment points around the articular surface remain intact^34,35^. It is reproducible about the distribution of articular surface fracture lines in the distal radius^36,37^. In 2011 and 2013, Gregory's team successively studied the relationship between the attachment of the ligament around the distal radius and the position of intra-articular fractures^36^. In this study, the periphery of the distal radius was divided into 11 regions (Fig. 11). The following regions are easily affected: the familiar site of the fracture was in the center of the sigmoid notch (inter-ligamentary region 10), the short radiolunate ligament, SRL), long radiolunate ligament (LRL) (ligament area 2), the dorsal center of the scaphoid fossa (ligament area 6), LRL (ligamentous area 3), and ulnar side of the dorsal scaphoid fossa (Ligament area 7). The following areas are less involved: dorsal ulnar radial ligament attachment (ligament area 9), palmar ulnar radial ligament attachment (ligament area 11), SRL attachment (ligament area 1), radial scaphoid ligament attachment (ligament area) 4), the radial the scaphoid fossa (ligament area 5), and the dorsal radiaisl wrist ligament attachment (ligament area 8)^36^. Fractures are more likely to occur in areas 2, 6, 10 than other areas, and there are significant statistical differences. Some scholars carried out atlas analysis of 40 cases of intra-articular fractures of the distal radius, and found that the characteristics of the horizontally oriented fracture line, which are the fracture line started from the base of the radial styloid process and extends horizontally along the scaphoid fossa and the lunar fossa to the sigmoid Trace middle^33^.

The three-column typing theory of Rikli and Regazzoni emphasizes the importance of the intermediolateral column. It consists of sigmoid notches and lunar fossa. When the wrist joint is under axial stress, the intermediate column load is higher than 50% of the axial pressure^31^. At the 0° position of the wrist joint, the matching area of the sigmoid notch and the ulnar head's articular surface is the largest. When the forearm is pronated or supinated, the matching area of the two decreases. At the junction of the sigmoid notch of the triangular fibrocartilage origin and the lunar fossa, the stops are widely attached to the triangular bone, the hook bone, etc., forming a stable structure of the lower ulnar radius and the ulnar side of the carpal bone^28^. If the fracture line involves the sigmoid notch, the distal radioulnar joint will be damaged, which changes the biomechanics and movement trajectory of the wrist and forearm. Post-traumatic sigmoid notch healing due to steps or gaps may cause wrist pain, instability, or loss of range of motion^29^. Therefore, it is particularly important to emphasize the reduction of the intermediolateral column. Among them, restoring the anatomical structure of the Distal radioulnar joint, especially the anatomical reduction of the sigmoid notch, is very important.

Melone focuses on and supplements the classification of the carpal surface of the distal radius. This classification emphasizes the importance of lunar fossa and its clinical prognosis, and include the formation mechanism of die-punch fractures analyzation. This classification benefits to identify surgical indications for intra-articular fractures and guide of intraoperative reduction and fixation, but it is useless in classifying extra-articular fractures. It is difficult to judge the four characteristic fracture blocks of the distal radius and the die-punch bone blocks based on X-rays alone without extensive experience. There is no satisfactory result on the observer consistency study of Melone typing. Melone classification includes five types based on the dorsal medial bone mass on the articular surface, the medial volar bone mass, the radial styloid bone mass, and the radial shaft. There may be some differences and variations in the four parts of the articular surface of the distal radius according to our judgment, in which the dorsal part of the scaphoid fossa and the lunar fossa are collectively summarized in the dorsal part of the 123 classification. Based on many studies on the characteristics of the fracture of the radial surface of the articular surface and the fracture of the articular surface, this study found that the dorsal part of the scaphoid fossa and the lunar fossa could be damaged at the same time due to the back extension of the wrist, was also verified in the analysis of patient imageology data^33–36^.

### Significance of 123 classification system for incision selection, reduction, and fixation

Because there is a stable radial wrist ligament between the lunar bone and the palamris or dorsal part, subluxation of the wrist joint is in 1-D and 1-V fracture. In this case, it should be fixed firmly by surgery. For 1-D fractures, the dorsal approach is preferred for reduction and fixation. If Henry approach is chosen, it will not only increase the difficulty of restoration but also may result in poor restoration and fixation strength due to failure to reset, particularly in lateral collapse. The dorsal approach uses a straight incision between the third and fourth extensor compartments, placing the steel plate in the fourth extensor compartment.

Similarly, 1-V fracture require a palmar approach for reduction and fixation. If a dorsal approach is chosen, it will not only increase the difficulty of reduction but may also lead to poor restoration. When the palmar part and the dorsal part are involved at the same time, the palmar approach is preferred for reset. For cases where the dorsal part cannot be reset through the palmar approach, a dorsal approach can be selected to assist in reset.

Rikli et al. confirmed in vitro studies that during regular wrist movements, the lunare fossa and radial sigmoid notch were subjected to > 50% axial pressure^38^. The level of CT can be used to intuitively and accurately determine the number of fracture lines passing through the lunare fossa. When the fracture line of the radial sigmoid notch passes, the radial sigmoid notch should be reduced and fixed first to avoid the abnormal healing of the radial sigmoid notch. While restoring the anatomical restoration and stable fixation of the sigmoid notch, it is necessary to take into account the stable fixation of the palmar and dorsal columns to avoid secondary displacement caused by a weak reset or poor fixation of the palmar or dorsal columns. A slight subluxation of the near-wrist carpal bones, thereby ensuring the restoration of the kinematics of the wrist joints.

### Advantages and disadvantages of the 123 classification system

This study is a retrospective study. All the included cases originated from inpatients, severe fractures in many patients. It is inevitable on case selection bias and statistical bias. Besides, the number of cases of some types is small due to incomplete image data of some patients, and the included research subjects are mainly inpatients. The larger sample size is necessary for practice and validation in the future. At the same time, more retrospective and prospective studies are necessary to verify the effectiveness of the 123 classification system in order to help better the surgeon understand the type of fracture in clinical work, and then formulate a surgical plan to obtain the desired results and judge the prognosis. Moreover, no control group treated non-operatively exists, constitutes another weakness of this study. Last, we could not exclude residual confounding by unmeasured or unknown confounders.

However, the CT-based 123 typing system provides a new classification idea. Based on the advantages of CT classification, the number of fracture lines, and the direction of bone mass displacement can be intuitively judged, which has a unique advantage for understanding fracture types and making surgical plans. At the same time, it benefits to improve the accuracy of typing, so it has better observer consistency. Besides, the 123 typing system is comprehensive and easy to remember. Of course, the traditional and conventional X-ray examinations cannot be replaced, and have certain reference functions for CT typing.

## Conclusion

The new classification system of distal radius fracture based on CT has theoretical and practical significance for selecting incision, guiding fracture block reduction and internal fixation. 123 classification system has clear classification, comprehensive coverage, easy to understand and remember. In addition, compared with other reported systems, it has ideal observer consistency results.

## Data Availability

The data used to support the findings of this study are included within the article. All data and materials were in full compliance with the journal's policy.
